# Assessing the burden of dengue: an important step toward committing resources to address this global disease

**DOI:** 10.2478/abm-2021-0025

**Published:** 2021-10-29

**Authors:** Editorial Office of Asian Biomedicine

**Affiliations:** Faculty of Medicine, Chulalongkorn University, Bangkok 10330, Thailand

Dengue is an important health issue worldwide. It is particularly widespread throughout the tropics including South-East Asia, Western Pacific, Eastern Mediterranean, Central America, and Africa. These are regions with much rainfall, tropical temperature, urbanization, and the presence of the principal mosquito vector *Aedes aegypti* and secondary vector *Aedes albopictus* [1].

Unreported and unrecognized apparent dengue virus infections make it difficult to estimate the true extent of the burden of dengue, making it difficult to track the progress of control measures needed to inform health policy [2]. Therefore, the global burden of the disease is not well known, but its epidemiological patterns can be high for both human health and the global economy [2].

Approaches to prevent dengue infection in endemic areas include mosquito control and vaccine development. Mosquito control is effective, but is difficult to sustain because the measure requires regular efforts of key participants [3, 4].

Developing an effective vaccine against dengue is challenging because the dengue virus has 4 serotypes (DENV-1 to -4), all of which can cause disease. Adverse reactions can be induced by pre-existing antibodies against primary infection with one type of dengue virus and subsequent infection with a different serotype. This means that neutralizing antibodies need to be generated to all serotypes of dengue to confer adequate protection [5]. To date, Dengvaxia (Sanofi Pasteur) tetravalent chimeras with yellow fever is the only vaccine licensed by European Union and United States regulatory authorities for use in seropositive individuals, but implementation has had limited to the Philippines and Brazil, at least until 2019 [6]. If given in seronegative individuals, there can be an increased risk of developing severe dengue disease [7]. Therefore, other possible dengue vaccines have been explored.

Children in dengue endemic areas are prone to develop severe dengue infections if they have a primary infection with DENV-3 and subsequent infection with DENV-2, -3, or -4 [8]. With no effective dengue vaccine for the general population or any antiviral therapy, dengue control continues to rely heavily on vector control measures, which are effective but difficult to sustain [3, 4]. Therefore, early and accurate diagnosis is important for guiding appropriate management, particularly, the occurrence of severe dengue infection and dengue shock syndrome.

Early recognition of severe disease and patients at increased risk of severe disease is important for their prompt treatment, with more aggressive therapy when warranted. Patients with a presumed diagnosis of dengue can be treated on an out-patient basis. They can be instructed to take plenty of fluids and watch for signs of dehydration. Inpatient management is warranted for patients with dengue and warning signs of severe infection, or dengue infection with coexisting conditions, shock, or other complications.

Capeding et al. sought to prospectively estimate the burden of dengue at the community level in an endemic area in the Philippines [9] in 500 participants, aged from 6 months to 50 years from various households, using an active sentinel surveillance strategy to determine the prevalence of dengue infection. Weekly surveillance to estimate the occurrence of virologically confirmed cases of dengue was used to follow up participating households. The prevalence of IgG for dengue was high, and some participants had dengue virus present at the time of active surveillance. The investigators sought to characterize the entire clinical spectrum of dengue infection as well as the transmission rate in the sentinel community. The information is important to encourage community participation in mosquito control and to prepare resources to meet the burden of patients across the clinical spectrum with a special focus on severe dengue disease. Severe disease can be common in dengue endemic areas where secondary dengue infection with different serotypes from the primary infection can be expected.

Until an effective vaccine or antiviral medication is widely available for common use, understanding the nature and extent of dengue problems in endemic areas is a vital weapon in our armamentarium to encourage public participation in mosquito control. Stakeholders have to combine their effort toward vector control measures and vaccine development [10]. This is particularly important with the current trends toward urbanization, scarce water supplies, and environmental changes. All these factors are conducive to an increasing trend of dengue infection.
